# Refractile bodies of *Eimeria tenella* are proteinaceous membrane-less organelles that undergo dynamic changes during infection

**DOI:** 10.3389/fcimb.2023.1082622

**Published:** 2023-03-09

**Authors:** Alana Burrell, Virginia Marugan-Hernandez, Karolin Graefin Von Der Recke, Kelsilandia Aguiar-Martins, Heloisa Berti Gabriel, Fiona M. Tomley, Sue Vaughan

**Affiliations:** ^1^ Department of Biological and Medical Sciences, Oxford Brookes University, Oxford, United Kingdom; ^2^ Department of Pathobiology and Population Sciences, The Royal Veterinary College, University of London, North Mymms, United Kingdom

**Keywords:** *Eimeria tenella*, refractile bodies, endogenous development, proteinaceous membrane-less organelles, actin dynamics, SBF-SEM

## Abstract

**Introduction:**

Refractile bodies (RB) are large membrane-less organelles (MLO) of unknown function found as a prominent mismatched pair within the sporozoite stages of all species of *Eimeria*, parasitic coccidian protozoa.

**Methods:**

High resolution imaging methods including time-lapse live confocal microscopy and serial block face-scanning electron microscopy (SBF-SEM) were used to investigate the morphology of RB and other intracellular organelles before and after sporozoite invasion of host cells.

**Results:**

Live cell imaging of MDBK cells infected with *E. tenella* sporozoites confirmed previous reports that RB reduce from two to one post-infection and showed that reduction in RB number occurs *via* merger of the anterior RB with the posterior RB, a process that lasts 20-40 seconds and takes place between 2- and 5-hours post-infection. Ultrastructural studies using SBF-SEM on whole individual sporozoites, both pre- and post-host cell invasion, confirmed the live cell imaging observations and showed also that changes to the overall sporozoite cell shape accompanied RB merger. Furthermore, the single RB post-merger was found to be larger in volume than the two RB pre-merger. Actin inhibitors were used to investigate a potential role for actin in RB merger, Cytochalasin D significantly inhibited both RB merger and the accompanying changes in sporozoite cell shape.

**Discussion:**

MLOs in eukaryotic organisms are characterised by their lack of a membrane and ability to undergo liquid-liquid phase separation (LLPS) and fusion, usually in an actin-mediated fashion. Based on the changes in sporozoite cell shape observed at the time of RB merger together with a potential role for actin in this process, we propose that RB are classed as an MLO and recognised as one of the largest MLOs so far characterised.

## 1 Introduction

Parasites of the genus *Eimeria* belong to the phylum Apicomplexa, which includes many important pathogens of humans and animals. All species within the phylum are parasitic protozoa that possess a specialised set of cytoskeletal and secretory organelle structures known as the apical complex (Okamoto and Keeling, 2014). Through histological examination of infected tissues, it was discovered that the lifecycle of *Eimeria* species involved release of invasive infectious stages from a spore (now known as the oocyst), replication within intestinal cells of the infected host, with formation of large multinuclear intermediate stages through a process known as schizogony, generation of dimorphic sexual stages followed by fertilisation and the formation of oocysts, and additional exogenous oocyst development following release from the host (Fantham, 1910). Other apicomplexan organisms, including *Toxoplasma gondii, Neospora caninum* and species of *Sarcocystis*, were found to share similar morphological features and lifecycle traits and these are all now grouped within the subclass of Apicomplexa known as the coccidia (Coccidiasina, (Hutchison et al., 1970; Hammond and Long, 1973).

Focusing on the impact of *Eimeria* species, the most clinically and economically relevant disease caused by this genus is poultry coccidiosis, an acute enteritis that induces clinical signs ranging from reduction in feed conversion and weight gain to severe diarrhoea with malnourishment, dehydration, and death, with estimated global costs of >£10 billion per annum (Blake et al., 2020). Chickens become infected after ingestion of oocysts from contaminated litter which release motile sporozoites that actively invade enterocytes, forming membrane-bound parasitophorous vacuoles within which parasite endogenous development occurs (Jensen, 1975; Russell, 1983). Sporozoites of *Eimeria* spp. contain most features of a eukaryotic cell as well as specialised apicomplexan organelles linked to their invasive parasitism, such as micronemes, rhoptries and an apicoplast (Burrell et al., 2020). Most prominently, they also contain two large cytoplasmic organelles known as refractile bodies (RB) (Ferguson et al., 1978).

Histochemical investigations of *Eimeria stiedae* revealed that RB are lipid rich (Frandsen, 1970) and proteomic investigation of RB constituents found them to contain several enzymes including an aspartyl protease (eimepsin; (Jean et al., 2000) and a nucleotide transhydrogenase, along with an array of proteins with unknown function (de Venevelles et al., 2006). These findings have led to hypotheses that RB could be nutrient stores or involved in metabolising energy reserves (Chobotar and Scholtyseck, 1982; Vermeulen et al., 1993; de Venevelles et al., 2006; Lemgruber and Lupetti, 2012). Interestingly, a family of proteins stored in the RB, including SO7 (also known as GX3262, RB1 or B antigen), induce immune-protective responses in the chicken and are potential candidates for inclusion in recombinant-based vaccines for coccidiosis (Crane et al., 1991; Kopko et al., 2000; Song et al., 2013).

A feature of *Eimeria* RB is that they lack a limiting membrane and have no discernible internal structures that can be visualised when chemically fixed and viewed by electron microscopy (Roberts and Hammond, 1970). Several microscopic investigations in different species of *Eimeria* have described RB undergoing dynamic changes throughout the endogenous parasite lifecycle, with sporozoites appearing to lose their anterior refractile body (RBant.) after invasion through a process that could be fragmentation, shrinkage or aggregation with the posterior refractile body (RBpost.) (Clark and Hammond, 1969; Fayer and Hammond, 1969; Roberts et al., 1970). As schizogony progresses, remaining RB material coalesces to a central position in the schizont and eventually disperses, with a small residue of refractile material incorporated into each daughter merozoite (Danforth and Augustine, 1989).

These behaviours of *Eimeria* RB are reminiscent of those exhibited by membrane-less organelles (MLO) (Nott et al., 2015; Uversky, 2017; Zaslavsky et al., 2018) also referred to as droplet organelles (Courchaine et al., 2016) or biomolecular condensates (Banani et al., 2017). MLOs are found in many eukaryotic cells, usually composed of proteins, RNA, and lipids that phase separate into condensed liquid states without a limiting membrane. They have physical properties described as a liquid-like behaviour, being able to drip, fuse, wet, and relax to spherical structures upon fusion (Darling et al., 2018).

We have used fixed and live imaging of intracellular parasites to investigate the dynamics and timing of RB merger and serial block-faced scanning electron microscopy (SBF-SEM) (Peddie and Collinson, 2014; Hughes et al., 2017) to compare the volumes of *Eimeria tenella* organelles before and after sporozoite invasion of cultured epithelial cells. Using these approaches, we were able to image in detail the dynamic changes that occur in RB organisation during the first few hours of intracellular parasitism. Using actin inhibitors, we discovered a potential role for actin in RB merger and propose that RB are large membrane-less organelles with MLO characteristics.

## 2 Results

### 2.1 Merger of the anterior refractile body (RBant.) occurs from two hours following infection, *via* a merging event with the posterior refractile body (RBpost.)

**Figure 1:**
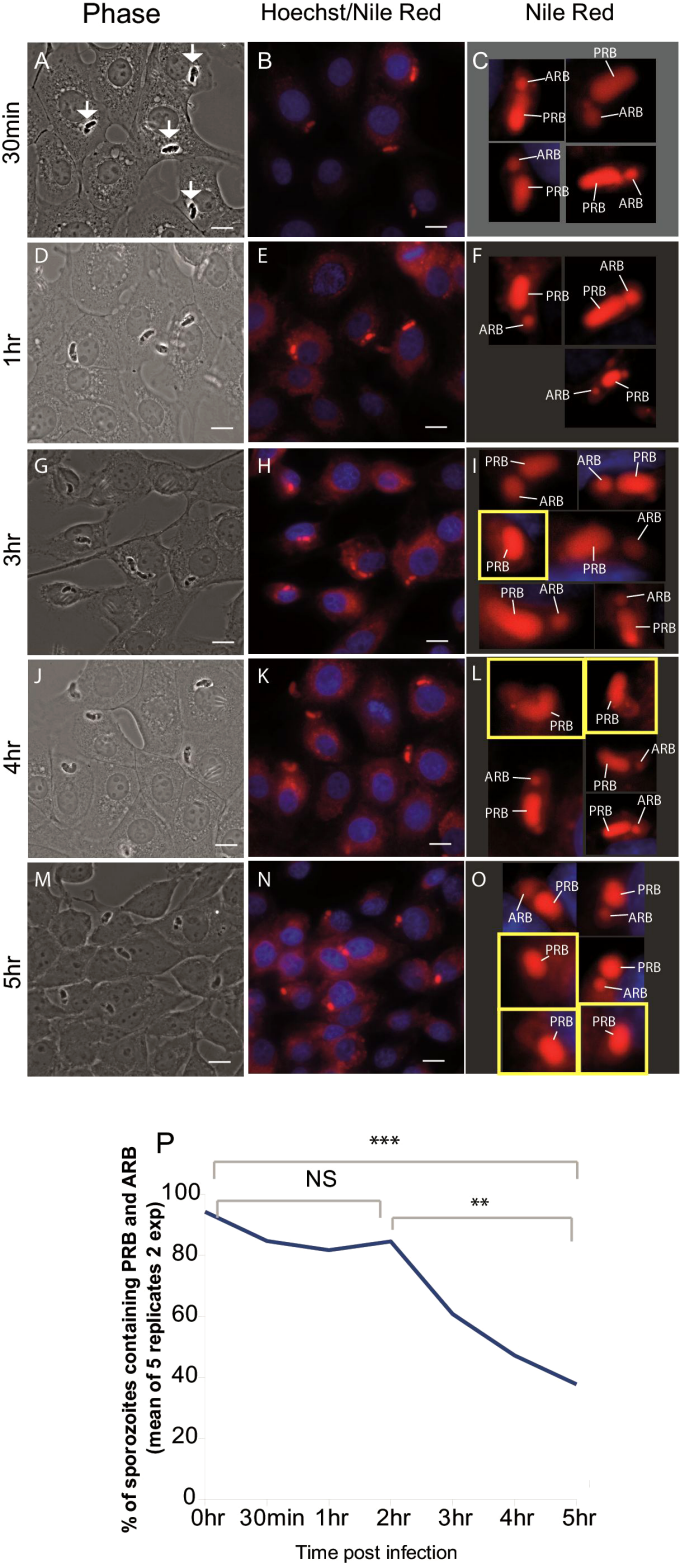
Loss of the anterior refractile body following host cell infection. Left panels **(A, D, G, J, M)** phase contrast light microscopy of sporozoites (arrows) within MDBK cells. Middle panels **(B, E, H, K, N)** Nile Red staining (red) localises to the RBant. and RBpost., Hoechst staining localises to host cell nuclei (blue). Right panels **(C, F, I, L, O)** magnification of single sporozoites visualised in the middle panels. Sporozoites containing a single refractile body are highlighted in a yellow square; **(P)** percentage of parasites containing both the RBant. and RBpost. from 0 hours (freshly hatched) to 5 hpi (two experiments/5 replicates) plotted to illustrate trend of RBant. reduction over the time course. Scale bars = 10 μm. **p ≤ 0.01, ***p ≤ 0.001; NS, not statistically significant.

To measure accurately when RB merger occurs, MDBK cells were infected with freshly purified sporozoites, fixed at different time points (0.5, 1, 2, 3, 4 or 5 hours post infection (hpi)) and stained with Nile Red and Hoechst, which allowed the visualisation of RB (red) and host-cell nucleus (blue); respectively (Hamid et al., 2015) (**Figure 1**). Two RB were readily detected in 96% and 92% of freshly-purified sporozoites (extracellular). Between 0-2 hpi this decreased to 78% and 93% (p=0.04 p > 0.05, T-test) and between 2-5 hpi to 22% and 56% (p=0.001 p=0.009, T-test) respectively (**Figure 1P**). For freshly-purified sporozoites incubated in DMEM alone, two RB were seen in 94%, 98% and 92% of cells at 0, 3 and 5 hours respectively, confirming that RB merger only occurs after invasion of host cells from 2 hpi onwards.

**Figure 2:**
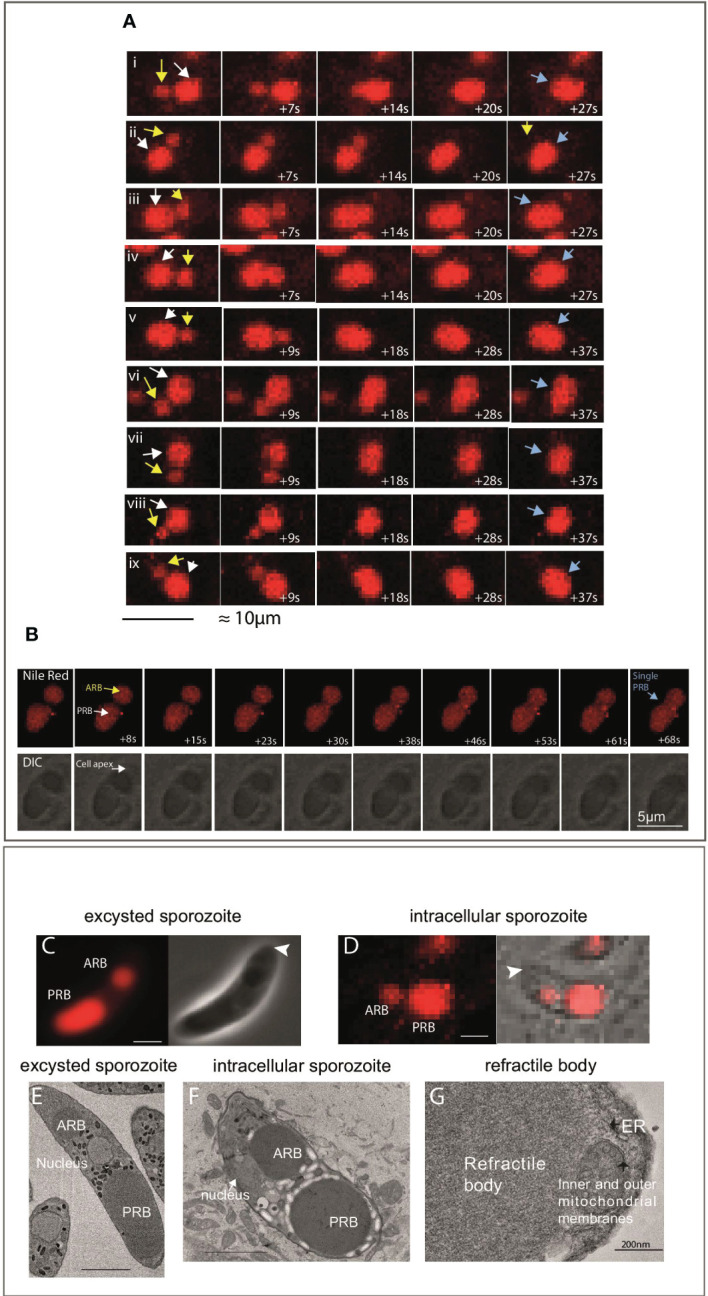
Live imaging of sporozoite refractile body merger. **(A)** Example of time-lapse events of RBant. loss seen in samples imaged between 2-4 hpi. The observed mechanism of RBant. loss was fusion/merger with the RBpost. The first time point shows a smaller RBant. (yellow arrow) positioned close to the large RBpost. (white arrow). In under 40 seconds, the RBant. has completely fused with the RBpost., leaving the sporozoite with a single RB (blue arrow) in the last panel (40x magnification). Scale bar 10 μm. **(B)** Top panel: fluorescent microscopy data of a single event of RB merger captured at 63x magnification. Bottom panel: DIC of the same events. Nile Red staining (red) localises to the RBant. and RBpost. Scale bar 5 μm. **(C)**: Phase contrast and fluorescence microscopy of a freshly-purified extracellular sporozoite stained with Nile Red, where there is an ~2 μm gap separating the RBant. with the RBpost. (arrowhead indicates cell apex). **(D)**: DIC and fluorescence microscopy of an intracellular sporozoite stained with Nile Red, where there is less than a ~1 μm gap separating the RBant. with the RBpost. (arrowhead indicates cell apex). **(E)**: SBF-SEM slice of a freshly-purified extracellular sporozoite to illustrate the positioning and gap between the RBant. and RBpost.; **(F)**: SBF-SEM slice of a intracellular sporozoite at 3 hpi. The RBant. is located alongside the nucleus and closer to the RBpost. This is not seen in freshly-purified extracellular sporozoite samples. **(G)** TEM showing an amplified region of a RB, no limiting membrane is observed.

To visualise this in living cells, MDBK cells were incubated with freshly-purified sporozoites and Nile Red in a temperature/gas-controlled chamber on a Zeiss LSM 880 AxioObserver microscope. Nineteen observations of RB merger were recorded, in all cases the RBant. (**Figure 2A**; yellow arrow in left panels) moved towards the RBpost. and a merger/fusion event occurred which took between 20-40 seconds (**Figure 2A**i-ix for examples). For each cell, the final panel (right) shows a time point where only a single RB is present (**Figure 2A**i-ix; blue arrows; **Supplementary Movie 1**). Time lapse was also carried out at a higher magnification (**Figure 2B**). In the first few frames, up to 23 seconds, both an RBant. (**Figure 2B**, yellow arrow) and a RBpost. (**Figure 2B**, white arrow) can be seen clearly, but by ~40 seconds only a single RB can be observed (**Figure 2B**; blue arrow). In all of the live cell imaging data, no events of RBant. loss *via* dissolution, shrinkage or fragmentation of either RB were observed (see Supplementary Video 1 for an example of the merger).

Careful examination of live cells (**Figures 2C, D**) and SBF-SEM (**Figures 2E, F**) of samples fixed at 0 or 3 hpi also support that the merger/fusion event involves the rearwards migration of the RBant. The RBant. was positioned laterally alongside the nucleus in the transmission electron microscopy (TEM) image of an intracellular sporozoite, close to the RBpost. (**Figure 2F**), in contrast to RBant. in extracellular sporozoites, which is always positioned anterior to the nucleus (**Figure 2E**). TEM also confirmed that RB possess no discernible limiting membrane (**Figure 2G**).

Taken together, our results establish that the RBant. merges with the RBpost. between 2-5 hours of host cell invasion, which is the total time frame used for the observations.

### 2.2 Reconstruction of individual parasites reveals major morphological changes during the first few hours after infection

**Figure 3:**
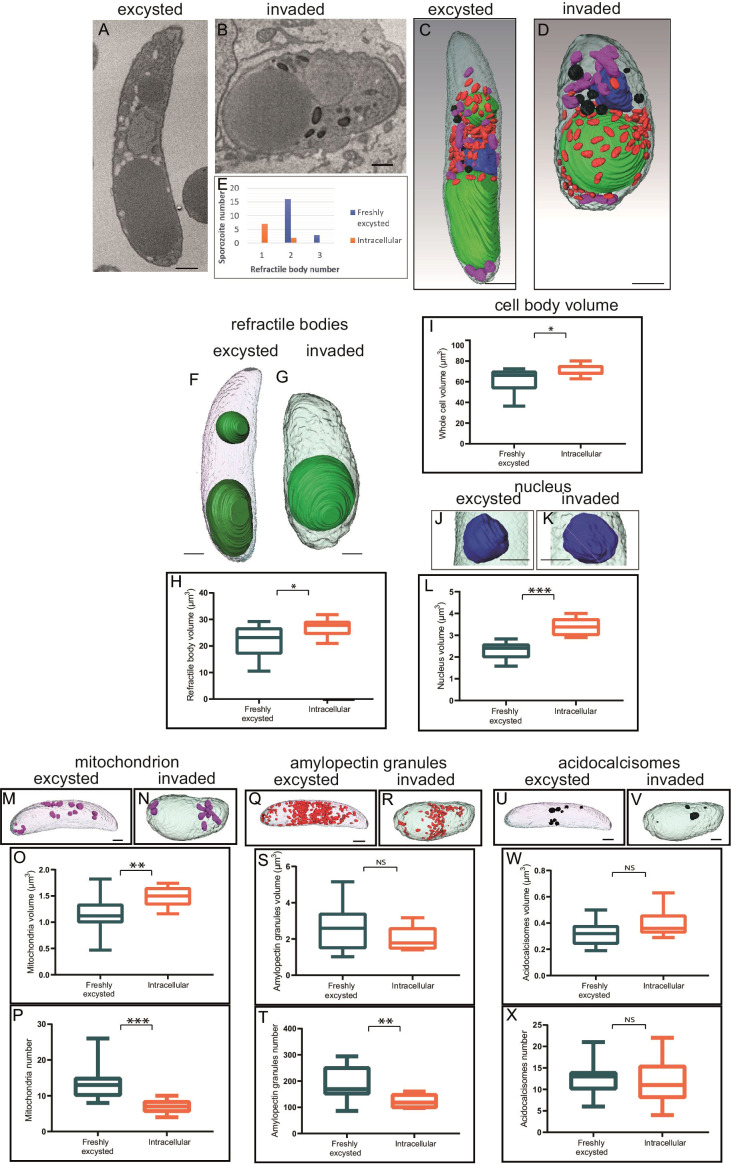
Morphometric comparison between extracellular and invaded sporozoites 2.5 hpi. **(A, B)** Single slice from SBF-SEM data of an extracellular sporozoite and an intracellular sporozoite at 2.5 hpi, respectively. **(C, D)** Three-dimensional model from SBF-SEM data of a extracellular sporozoite and an intracellular sporozoite at 2.5 hpi, respectively. Nuclei (blue), RB (green), amylopectin granules (red), mitochondria (purple) and acidocalcisomes (black); Scale bars ~ 1 μm. **(E)** Numbers of extracellular or intracellular (2.5 hpi) sporozoites containing either one, two or occasional three RB; **(F-X)** Morphometric comparison between extracellular and intracellular sporozoites for RB volume **(F-H)**, cell body volume per cell **(I)**, nucleus volume per cell **(J-L)**, mitochondria volume and number per cell **(M-P)**, amylopectin granule volume and number per cell **(Q-T)**, acidocalcisome volume and number per cell **(U-X)**. NS = not significant, *p ≤ 0.05, **p ≤ 0.01, ***p ≤ 0.001 – T test or Mann Whitney U for volume measurements. Scale bars = 1µm; NS, not statistically significant.

Reconstruction of whole individual extracellular sporozoites by SBF-SEM were originally published (Burrell et al., 2022) and is used in this study to compare with intracellular sporozoites (2.5 hpi). **Figures 3A–D** are example slices from SBF-SEM data and whole cell segmentation of the major organelles identified in extracellular (**Figures 3A, C**) and intracellular sporozoites (**Figures 3B, D**). There were two striking morphological differences between extracellular and intracellular sporozoites. Firstly, intracellular sporozoites are significantly shorter and rounded up compared to extracellular sporozoites (**Figures 3A–D**). Secondly, there is a reduction in the number of RB from two in extracellular sporozoites to one in intracellular sporozoites (**Figures 3E–G**) (in agreement with previous observations reported by Fayer and Hammond, 1969), consistent with our light microscopy data using Nile Red.

The detailed SBF-SEM datasets revealed that the mean total volume of the single RB in intracellular sporozoites was significantly greater, at 26.9 µm³, than the mean total volume of RBant. and RBpost. together in extracellular sporozoites, at 22.1 µm³ (p=0.02, T-test) (**Figures 3F–H**). The increase in overall volume of the single RB in intracellular sporozoites supports a merging of the two RB rather than loss of the RBant. following host cell invasion *via* a breakdown of the organelle within the cell.

The SBF-SEM data also revealed other alterations of the intracellular sporozoite compared to extracellular sporozoites. The whole cell volume increased significantly post-invasion from 61.3 µm³ to 70.6 µm³ (**Figures 3C, D, I**). To determine if change in cell shape correlated with RB merger, sporozoite lengths and widths were measured at 5 hpi by light microscopy and scored for presence/absence of the RBant. using Nile Red. Mean length divided by width value (length/width) of sporozoites before merger and containing an RBant. was 2.41 whereas mean length/width of sporozoites after merger and without an RBant. was 1.95 (p = 0.013, T-test) (**Supplementary Figure 1**). These data suggest that the extent of sporozoite rounding (greater roundness having length/width value closer to one) is associated with RB merger.

The volume of the nucleus increased from 2.3 µm³ to 3.4 µm³ in intracellular sporozoites (**Figures 3J–L**) and mitochondrial volume (**Figures 3M–O**) increased from 1.1 µm³ to 1.6 µm³ in intracellular sporozoites. However, the number of mitochondria decreased from an average of 14 to 7 post-invasion (**Figure 3P**). No significant differences were observed for the overall volume of amylopectin granules (**Figures 3Q–T**) and acidocalcisomes following invasion (**Figures 3U–X**), but there was a significant decrease in amylopectin granule number post-invasion (**Figure 3T**). None of the observed morphological changes were seen in extracellular sporozoites incubated in medium without cells for a 2.5 hours period, indicating that they are linked to the processes of intracellular parasitism. These data suggest that the morphological differences seen between extracellular sporozoites and intracellular sporozoites are dependent on sporozoites entering host cells.

### 2.3 Treatment of infected cell monolayers with actin inhibitors reduce RB merger in intracellular sporozoites

**Figure 4:**
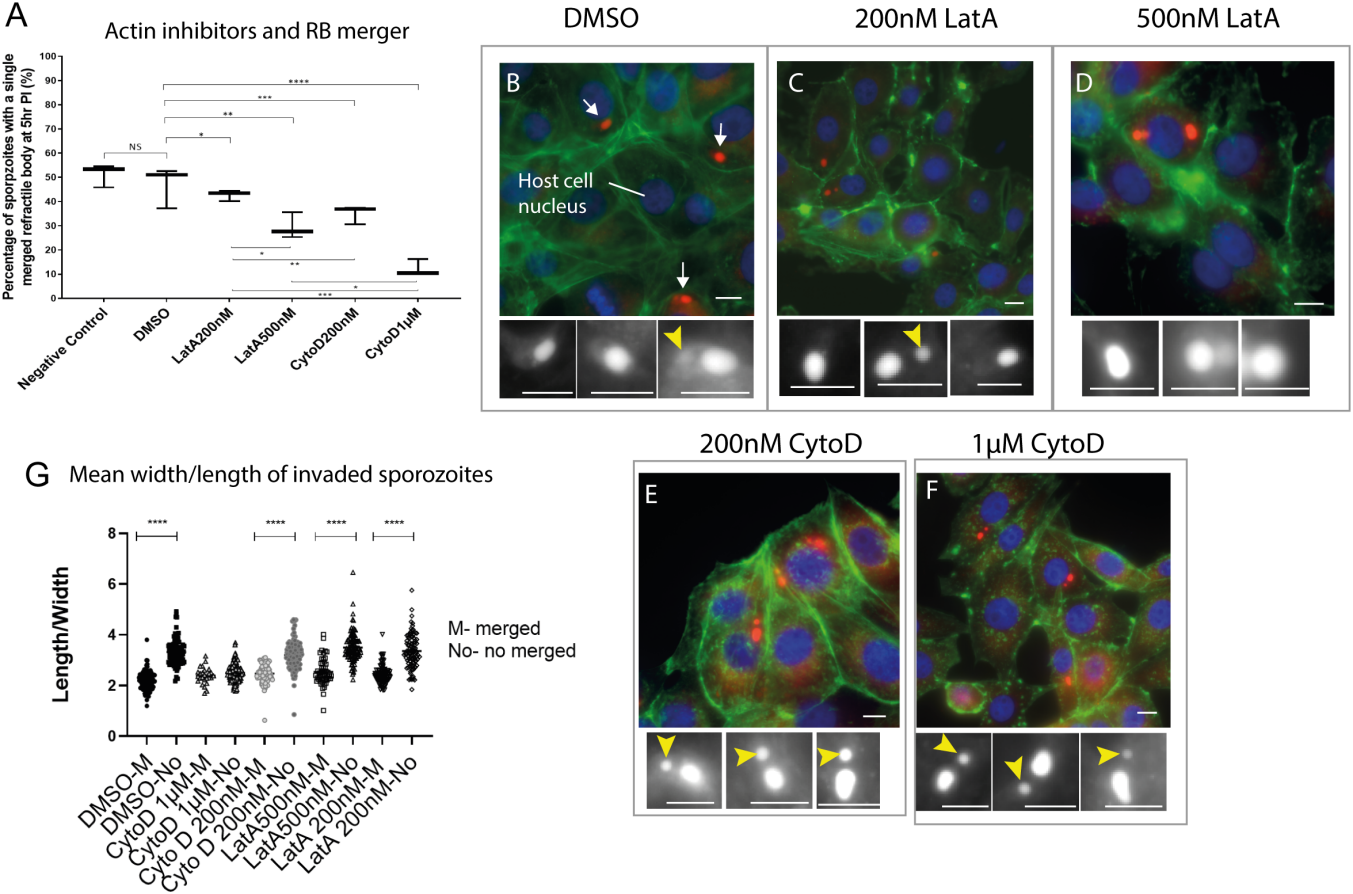
Effects of actin inhibitors on refractile body merger and morphology changes in intracellular sporozoites. **(A)**. Inhibition RB merger following treatment with actin inhibitors. DMEM - negative control, DMSO only control, 200 nM Latrunculin A (LatA), 500 nM Latrunculin A, 200 nM Cytochalasin (CytD) or 1µM Cytochalasin D; NS = not significant, *p ≤ 0.05, **p ≤ 0.01, ***p ≤ 0.001 - One Way ANOVA and *post-hoc* Turkey test) (n=100 cells/treatment). **(B–F)** Light microscopy data of sporozoites (arrows) within MDBK cells incubated in the presence of DMSO, 200 nM Latrunculin A, 500 nM Latrunculin A, 200 nM Cytochalasin or 1 µM Cytochalasin D. For each condition, sporozoites were assessed for the presence of an RBant. (yellow arrowhead). Nile Red staining highlights the RB (red) (white in the cropped images), Hoechst staining localises to the host cell nucleus (blue) and Phalloidin 488 localises to the host cell actin (green); **(G)** Comparison of cell rounding following 5 hours incubation with each treatment. Scale bars = 20µm. ****p < 0.05.

Rearwards migration and merger of the RBant. to the RBpost. resembles the behaviour of membrane-less organelles (MLOs) and actin has been shown to be important in MLO dynamics (Feric and Brangwynne, 2013). Actin-disrupting toxins that have differential inhibitory effects on host or parasite actin filament polymerisation were used to evaluate potential effects in RB merger and shape rounding in intracellular sporozoites. In infected cells treated with DMEM alone or DMSO, sporozoite RB merger had occurred in 52% and 47% of sporozoites, respectively at 5 hpi (**Figure 4A**). At the same time point, in cells treated with Latrunculin A (LatA) 200 nanomolar (uM) or 500 nM, RB merger had occurred in 42% and 29% of sporozoites, respectively; and with Cytochalasin D (CytD) 200 nM or 1 micromolar (μM) this was reduced further to 35% and 12%, respectively. These results suggest an apparent dependency on actin polymerisation for RB merger.

As showed in the previous section (**Supplementary Figure 1**), RB merger correlates with the rounder shape of intracellular sporozoites. We therefore tested if the treatment with actin inhibitors could also affect changes in the shape of sporozoites. Measurements of parasite length and width were taken in intracellular parasites following treatment of infected cells with CytD, LatA or DMEM/DMSO controls. For all groups except CytD 1 μM, the change to a rounded parasite shape correlated with RB merger in line with what is observed in untreated control cells (**Figure 4G**). However, all intracellular sporozoites treated with CytD 1 μM were rounder than controls even if RB merger had not occurred, suggesting that the higher concentration of CytD causes parasites to round up in the absence of RB merger.

Finally, the state of actin filaments in host cells following treatments was noted (**Figure 4**, **Supplementary Figure 2**). Host cells in the diluent control (DMSO) and 200 nM CytD treatments contained long fibres, indicative of minimum damage in actin filaments however those treated with 200 nM LatA, 500 nM LatA and 1 μM CytD showed degrees of host cell actin filament disruption (**Supplementary Figure 2**). Thus whilst the experiments suggest a role for parasite actin in RB merger it is also possible that disruption to host cell actin following some treatments may affect intracellular parasite development and lack of RB merger is an incidental effect.

## 3 Discussion

Detailed morphometric measurements of extracellular and intracellular sporozoites taken from SBF-SEM three-dimensional models and light microscopic investigations of sporozoite shape and RB dynamics has revealed that *E. tenella* parasites undergo significant structural changes during the first five hours after invasion of host cells. In addition to a significant rounding up of intracellular sporozoites, the most notable feature is a reduction in the number of RB from two to one, achieved by a process of merger which resembles that reported for droplets of protein mixtures (Li et al., 2012). Taken all together, those observed alterations in sporozoites were associated with the host cell invasion event. Afterwards, during intracellular infection by *Eimeria* parasites, a multinuclear schizont forms in preparation for cell division and formation of merozoites. During schizogony the single merged RB disperses to 6-10 globules, which diffuse before re-concentrating as a small focus in each mature first-generation merozoite (Danforth and Augustine, 1989) again consistent with characteristics of MLO. Moreover, disruption of RB diffusion and dispersal by treatment with a monoclonal antibody to a RB protein inhibited schizont development (Danforth and Augustine, 1989). These discoveries, together with the overall morphology of RB and the absence of a limiting lipid membrane around these organelles are shared features with MLOs.

MLOs in eukaryotes include P granules and other germ granules, stress granules and P-bodies in the cytosol and nucleoli, paraspeckles, Cajal bodies, and PML bodies in the nucleus (Mitrea and Kriwacki, 2016). They are generally described as organelles that are not membrane-bound and that exhibit liquid-like characteristics which include a spherical shape, permeable surface, dynamic internal components, and ability to flow, drip, and fuse (Forman-Kay et al., 2018). MLOs often contain proteins that harbor multiple modular domains or stretches of low-complexity amino acid sequence with repeated interaction motifs or charged elements (Nott et al., 2015) as well as nucleic acids, typically RNA. Interestingly, the well-characterised RB protein SO7 as well as other RB proteins contain high levels of low complexity homo-polymeric amino acid repeats (Liberator et al., 1989; Reid et al., 2014), providing additional circumstantial evidence that the RB may be an MLO. Interactions between multivalent macromolecules in MLOs are critical for polymerization-driven phase separation that causes the formation of a condensed, droplet phase suspended in the bulk solution phase. Only a small number of these components appear to be essential for the structural integrity of the MLO (Clemson et al., 2009). Recently, the process of cellular phase separation has been associated with many additional, diverse biological structures including signaling centers formed by trans-membrane receptors (Su et al., 2016), membrane-bound protein assemblies that initiate endocytosis (Bergeron-Sandoval et al., 2021). In *Plasmodium* and other Apicomplexa, the crystalloid (Dessens et al., 2021), a cytoplasmic inclusion of similar morphology to the RB of *Eimeria* and *Lankesterella* spp., have been described. Nonetheless, little is known about the nature and role of these organelles.

Inspired by findings that MLO structures exhibit actin-dependent fusion dynamics (Feric and Brangwynne, 2013), we investigated the potential role of actin in RB merger. Apicomplexan actin is divergent from that of other eukaryotic organisms (Dobrowolski et al., 1997; Schmitz et al., 2005; Skillman et al., 2011) and to investigate its potential role in RB fusion, we selected two inhibitors that have differential effects on apicomplexan and host actin. The cell permeable toxin Cytochalasin D (CytD) is active against both parasite and host actin, binding to the barbed end of microfilaments causing their disruption and inhibiting polymerisation (Periz et al., 2017). Latrunculin A (LatA) binds host cell actin monomers, and blocks polymerisation, but has no effect on apicomplexan actin, therefore, LatA has no effect on eimerian parasites (Whitelaw et al., 2017). The specific role of parasite actin could be therefore inferred by evaluating the differential effect in intracellular sporozoites of host-parasite simultaneous actin inhibition caused by CytD compared to the effect of host-only actin inhibition caused by LatA. The use of LatA alongside CytD has previously been employed to determine whether phenotypic changes occur due to disruption of parasite actin, whilst controlling for effects on host cell actin (Periz et al., 2017).

It is well reported that when used to pre-treat extracellular sporozoites, CytD blocks *Eimeria* gliding motility and invasion (Bumstead and Tomley, 2000), therefore, to avoid unwanted effects on invasion, *E. tenella* sporozoites were left to invade host cells prior to treatment with different concentrations of CytD, LatA or diluent (DMSO) controls. We conclude that RB body merger is at least partially dependent on functional parasite actin but cannot rule out the possibility that host actin inhibition by these toxins may contribute to the reduction in merger of RB in sporozoites inside the parasitophorous vacuole within host cells.

We propose that RB merger and shape change are linked and offer some potential hypotheses. In extracellular *E. tenella* sporozoites, an array of microtubules is present beneath the plasma membrane and inner membrane complex (IMC) (Russell and Sinden, 1982). It is likely that the arrangement of these microtubules confers structural stability to the elongated shape of the sporozoite. For sporozoite rounding to occur at RB merger, the spatial arrangement of subpellicular microtubules and actin could require re-modelling and F-actin is associated with the subpellicular microtubules so it makes sense that any remodelling could occur in tandem (Periz et al., 2019). In *T. gondii* tachyzoites it has been shown that microtubules remain fixed in position at the apical polar ring (Frenal et al., 2017) thus following invasion, actin may be involved in constriction of parasite length, possibly by increasing intracellular pressure leading to passive spreading of the subpellicular microtubule cytoskeleton. Alternatively, actin may be directly associated with widening the spaces between the distal ends of the subpellicular microtubules. For an increase in sporozoite roundness to occur there may also be changes to the IMC. It is not yet clear whether this complex of alveolar sacs has enough flexibility to accommodate the observed changes to sporozoite shape, or whether IMC disruption occurs to permit sporozoite rounding. In *E. tenella* schizonts (cell undergoing nuclear division in many apicomplexan parasites) at ~35 hpi much of the IMC structure is lost (Pacheco et al., 1975), so it is possible that changes to the IMC are initiated during the first few hours after invasion. Finally, there could be detachment of the subpellicular microtubules from the surrounding pellicle membrane, which allows passage of cytoplasmic material into the space between the microtubule scaffold and sporozoite-surface pellicle. In this scenario, changes to sporozoite shape may occur without any changes to the shape of the microtubule scaffold.

The molecular mechanisms of merger could be a passive event with actin-driven changes in parasite cell shape leading to spatial re-organisation of the organelles. This could lead to closer association of the RB. Analyses of other MLOs have demonstrated that they exist due to phase-separation (Uversky, 2017), where two liquids do not readily mix with one another. If, as hypothesised, RB are maintained by phase-separation, contact between two droplets of the same nature/composition is likely to result in a fusion event. Alternatively, there could be a direct interaction between actin filaments and RB where actin filaments may attach to the RBant.; actin-filament polymerisation or actin-associated molecular motor interactions could therefore result in RBant. relocation and fusion with the RBpost. Current developments in genetic modification of *Eimeria* species (Pastor-Fernandez et al., 2019) could support future studies allowing the visualisation of actin filaments (Periz et al., 2017) and/or their disruption, that, together with microscopy analysis, could discern which processes are essential for RB merger, assess the hypothesis that RB play an essential role in parasite survival and how they could be targeted.

Finally, based on our understanding and observations of these structures, they fulfil the defined criteria and characteristics and would represent one of the largest MLOs discovered in eukaryotic cells at 22μm^3^ and taking up ~36% of the total cell volume of extracellular sporozoites (Burrell et al., 2022). We propose that the RB should be classified as an MLO.

## 4 Materials and methods

### 4.1 Parasites and birds

Three-week-old White Leghorn chickens reared under specific pathogen free conditions were used for the propagation of *E. tenella* Wisconsin strain (Shirley, 1995). Oocyst purification, excystation and sporozoite purification were performed as described before (Pastor-Fernandez et al., 2019).

### 4.2 Cell culture

The NBL-1 line of Madin-Darby bovine kidney (MDBK) cells (NBL-1; ECACC-Sigma-Aldrich, Salisbury, UK) were passaged twice weekly by trypsinisation of confluent monolayers and maintained in T75 (10x10^6^ cells/well) flasks at 37°C, 5% CO_2_ in Advanced DMEM (Gibco, Leicestershire, UK) supplemented with 2% foetal bovine serum (FBS; Sigma, Suffolk, UK) and 100 U penicillin/streptomycin (Fisher, Leicestershire, UK). For infections with sporozoites, MDBK cells were seeded onto 24-well plates (with or without 13mm diameter coverslips; specified for each experiment) and left to settle for 2 hours at 38°C – 5% CO_2_. Freshly-purified sporozoites were added at a specific ratios (sporozoites:cells) and incubated at 41°C – 5% CO_2_.

### 4.3 Serial block face – scanning electron microscopy (SBF-SEM)

MDBK cells were seeded at 0.35x10^6^ cells/well and after 2 hours freshly-purified sporozoites were added at a 10:1 ratio for 2.5 hours. Monolayers were released with 0.05% trypsin – 0.02% EDTA and suspended in 1 ml of primary fixative (2% freshly-prepared formaldehyde solution (Sigma-Aldrich), 2.5% electron microscopy grade glutaraldehyde (TAAB) and 0.1 M sodium cacodylate buffer (TAAB) in double distilled (dd)H2O for 2 hours at 4˚C. Cells were then washed in 0.1 M cacodylate buffer and incubated in 1.5% potassium ferrocyanide (AnalaR NORMAPUR ^®^) and 2% osmium tetroxide (TAAB) in 0.1 M cacodylate buffer for 60 minutes at 4°C. Following washes in ddH20, samples were incubated in 2% osmium tetroxide in ddH2O for 60 minutes at 4°C. After five washes in ddH20, samples were added to 1 ml molten 3% agarose and centrifuged. Agarose and infected cells were transferred to 4°C for 5 minutes to set. Approximately 1 mm³ blocks were cut and incubated in freshly-filtered 2% uranyl in the dark at 4°C overnight. Samples were washed in ddH20 then dehydrated by a series of 20 minutes incubations in acetone/ddH20 solutions. Dehydration steps consisted of increased concentrations of acetone from 30% to 100%. Samples were incubated for 2 hours in 25% epoxy resin (TAAB 812 resin premix kit) in acetone and then incubated in 50% resin in acetone overnight; 75% resin in acetone for 6 hours; 100% resin overnight and two changes of 100% resin for 2 hours each.

### 4.4 SBF-SEM image acquisition

SBF-SEM data was collected using a Merlin VP SEM (Zeiss) scanning electron microscope with 3view automated sectioning and image capture system (Gatan). Samples were trimmed to ~1 mm³ and mounted on 3view sample pins using an epoxy conductive adhesive from Circuitworks™. The block-face was imaged using a scanning electron beam with 3-5 kV accelerating voltage. Electron signal was detected using a back-scatter electron detector (OnPoint, Gatan) and nitrogen gas was injected to raise chamber pressure to 30pa. Pixel size was between 3-7 nanometer (nm) with a section thickness from 60-100 nm. Dwell time was approximately two seconds per pixel with a scan area of around 4,000 x 4,000 pixels. Completed SBF-SEM data sets contained anywhere from 50 – 500 sections.

### 4.5 SBF-SEM data processing and structure segmentation

SBF-SEM data was processed using IMOD (Kremer et al., 1996) software. Image files were converted from.dm4 format to.mrc data stacks. Voxel size was adjusted in Z to reflect the slice thickness used, *via* the command ‘alterheader’. Data was binned once (combining adjacent pixels to reduce the file size), floated (simplifying pixel values) using ‘newstack’ commands, smoothed (averaging of pixels with their neighbours), and flipped using ‘clip’ commands. The image stack was then subject to automated alignment through the IMOD align serial sections interface. Regions of interest were identified and trimmed using the command ‘trimvol’. Statistical analysis was performed using IBM™ SPSS™ version 25 software; T-test and Mann Whitney U for whole cell and organelle volume in a total of 13 intracellular sporozoites was applied. Quantification of extracellular sporozoites in **Figure 3** was published by (Burrell et al., 2022) from data of 25 sporozoites. This published data was used in this study to provide a comparison with the intracellular infective stage, which has not been previously published.

### 4.6 Quantification of sporozoite dimensions by light microscopy

Freshly-purified sporozoites were incubated for 6 hours in DMEM, and MDBK monolayers infected with sporozoites for 5 hours (0.1x10^6^ cells/well infected at a 0.5:1 ratio) were fixed with 4% buffered formaldehyde for 10 minutes and then washed in 0.1 M phosphate buffer. Image J software (NCBI, http://rsb.info.nih.gov/ij/) was used for sporozoite length and width measurements. Statistical analysis was performed using IBM™ SPSS™ version 25 software, One-Way ANOVA was applied followed by a *post-hoc* Turkey HSD test.

### 4.7 Quantification of anterior refractile body presence

MDBK monolayers infected with sporozoites for 0.5, 1, 2, 3, 4 and 5 hours (0.1x10^6^ cells/well infected at a 0.5:1 ratio) were fixed with 4% buffered formaldehyde for 10 minutes and then washed in PBS. Fixed and stained samples with Nile Red were viewed using a widefield fluorescence microscope (AxioImager, Zeiss). For each condition, between 100 and 200 sporozoites were assessed for the presence or absence of an RBant. (Nile Red). Intracellular location of sporozoites was confirmed by detection of adjacent host-cell nuclei (Hoechst). Brightfield was also recorded for evaluation of sporozite dimensions. Statistical analysis was performed using IBM™ SPSS™ version 25 software; T-test was applied.

### 4.8 Live cell imaging of anterior refractile body loss

For live cell microscopy, infected monolayers were prepared on a μ-Slide 2-well chambered coverslip (Ibidi) (0.5x10^6^ cells/well infected at a 1:1 ratio). The μ-Slide was placed onto the stage of a confocal microscope (LSM 880 AxioObserver – Zeiss) which was set at 41°C, 5% CO_2_. Nile Red (200 μg/ml in acetone) was added to each well. Fluorescence imaging was performed using continuous z-stack acquisition encompassing all cell-containing focal planes. Images were acquired for both differential interference contrast (DIC) and fluorescence. Imaging was performed using either a 40x objective (voxel size 0.42 micrometer (μm) x 0.42 μm x 1-2 μm) or a 63x objective (voxel size 0.26 μm x 0.26 μm x 0.59 μm). Each field was imaged for a maximum of 15 minutes to minimise thermal damage to the cells. Staining of free live sporozoites with Nile Red at different concentrations (0.5 μg/ml to 100 μg/ml) did not show significant differences in ABR presence, proving that Nile Red has no deleterious effect in sporozoites and does not affect RBant. loss.

### 4.9 Actin inhibition experiments

MDBK monolayers infected with sporozoites (0.3x10^6^ cells/well infected at a 10:1 ratio) were left for 30 minutes to allow invasion without interference of actin inhibitors. DMEM was the removed and CytD (1 μM/200 nM) or LatA (500 nM/200 nM) were added to three individual wells. DMSO and DMEM were also added as untreated controls. Following treatment, samples were incubated for 4.5 hours at 41°C - 5% CO_2_ then fixed in 4% formaldehyde. Samples were stained with Nile Red, Hoechst and Phalloidin 488. Digital images from fixed and stained infected monolayers were captured using a Leica DMI300 B microscope equipped with a high-speed camera DCF365FX. Images were used to assess the RBant. presence and the sporozoite dimensions using Image J as described above. Statistical analysis was performed using Graphpad Prism software v.7.0b; One Way ANOVA and *post-hoc* Turkey test was used.

### 4.10 Nile Red, Hoechst and Phalloidin 488 staining

Fixed sporozoites or infected monolayers were washed in PBS, incubated for 15 minutes in 0.1% Triton X-100 and washed again in PBS. Then incubated in 1% glycine dissolved in PBS and washed in PBS. Samples were stained with 5 μl of Nile Red (2 mg/l in PBS) for 15 minutes in the dark; and/or 1 μl Hoechst (5 g/l in PBS) for 15 minutes in the dark; and/or 10 μl of Phalloidin 488 (6.6 μM in methanol) for 45 min. Washes were done between staining and at the end with PBS.

### 4.11 Ethical statement

This study was carried out in strict accordance with the Animals (Scientific Procedures) Act 1986, an Act of Parliament of the United Kingdom. All animal studies and protocols were approved by the Royal Veterinary College Ethical Review Committees and the United Kingdom Government Home Office under specific project licences.

## Data availability statement

The original contributions presented in the study are included in the article/**Supplementary Material**. Further inquiries can be directed to the corresponding authors.

## Ethics statement

The animal study was reviewed and approved by Royal Veterinary College Ethical Review Committees and the United Kingdom Government Home Office.

## Author contributions

Conceived and designed the experiments: AB, VM-H, FT, and SV. Experiments performing: AB, VM-H, KG, K-AM, and HG. Analysis of data: AB, VM-H, KG, and K-AM. Manuscript writing: VM-H, SV, AB and FT. All the authors commented in the manuscript. All authors contributed to the article and approved the submitted version.

## Supplementary material

Supplementary Figure 1 Refractile body merger is associated with sporozoite roundness. **(A, B)**: Light microscopy data of a sporozoite with 2 RB fixed at 5 hpi Cell length (yellow) and cell width (cyan) were measured. The RBant. (arrowhead) visualised using Nile Red. **(C, D)**: Light microscopy data of a sporozoite fixed at 5 hpi where merger has occurred. Note the absence of RBant. in **(D)**. **(E)**. Comparison of length/width values for presence or absence of an RBant. at 5 hpi (n = 17). Mean length/width value for sporozoites with an RBant. was 2.41 compared to mean length/width value of 1.95 in sporozoites where merger has occurred (p = 0.013, T-test).

Supplementary Figure 2 Analysis of Host cell actin following treatment with actin inhibitors. Following Phallodin Atto 488 staining at 5hrpi, control host cells (DMSO) exhibited numerous intact actin filaments. Host cells treated with 200 nM CytoD, contained intact actin filaments and some granularity throughout the cytoplasm. Host cells treated with either 1µM CytoD or 200 nM LatA showed comparable actin staining patterns and both contained faint filaments, lower in density than for those treated with 200 nM CytoD or control, and had a granular pattern throughout the cytoplasm. Host cells treated with 500 nM LatA exhibited the most disruption to actin-filament structure. Scale bar ≈ 20µm

Movie 1 Live cell imaging of RB merger 63X. See methods for details.
