# Supplementary figures and images for: Refractile bodies of Eimeria tenella are proteinaceous membrane-less organelles that undergo dynamic changes during infection
Source: Front Cell Infect Microbiol. 2023 Mar 9;13:1082622. doi: 10.3389/fcimb.2023.1082622 (PMC10081493; doi:10.3389/fcimb.2023.1082622)

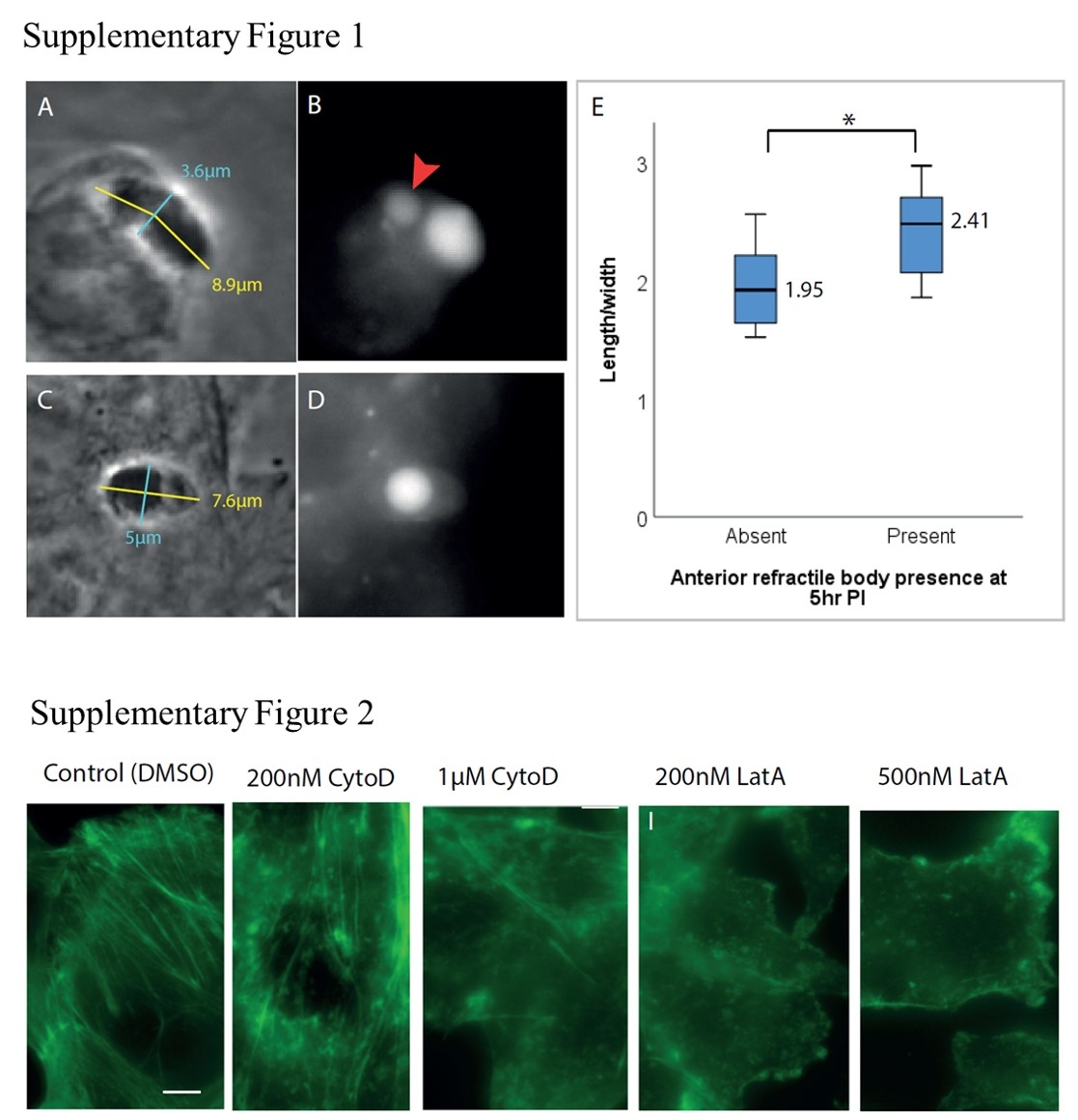

Supplement: Supplementary Figure 1 — Refractile body merger is associated with sporozoite roundness. (A, B): Light microscopy data of a sporozoite with 2 RB fixed at 5 hpi Cell length (yellow) and cell width (cyan) were measured. The RBant. (arrowhead) visualised using Nile Red. (C, D): Light microscopy data of a sporozoite fixed at 5 hpi where merger has occurred. Note the absence of RBant. in (D). (E). Comparison of length/width values for presence or absence of an RBant. at 5 hpi (n = 17). Mean length/width value for sporozoites with an RBant. was 2.41 compared to mean length/width value of 1.95 in sporozoites where merger has occurred (p = 0.013, T-test). [file Image_1.jpeg]
